# Long Term Influence of Carbon Nanoparticles on Health and Liver Status in Rats

**DOI:** 10.1371/journal.pone.0144821

**Published:** 2015-12-14

**Authors:** Barbara Strojny, Natalia Kurantowicz, Ewa Sawosz, Marta Grodzik, Sławomir Jaworski, Marta Kutwin, Mateusz Wierzbicki, Anna Hotowy, Ludwika Lipińska, André Chwalibog

**Affiliations:** 1 Division of Nanobiotechnology, Warsaw University of Life Sciences, Warsaw, Poland; 2 Department of Chemical Technologies, Institute of Electronic Materials Technology, Warsaw, Poland; 3 Department of Veterinary Clinical and Animal Sciences, University of Copenhagen, Copenhagen, Denmark; University of California, Merced, UNITED STATES

## Abstract

Due to their excellent biocompatibility, carbon nanoparticles have been widely investigated for prospective biomedical applications. However, their impact on an organism with prolonged exposure is still not well understood. Here, we performed an experiment investigating diamond, graphene oxide and graphite nanoparticles, which were repeatedly administrated intraperitoneally into Wistar rats for four weeks. Some of the animals was sacrificed after the last injection, whereas the rest were sacrificed twelve weeks after the last exposure. We evaluated blood morphology and biochemistry, as well as the redox and inflammatory state of the liver. The results show the retention of nanoparticles within the peritoneal cavity in the form of prominent aggregates in proximity to the injection site, as well as the presence of some nanoparticles in the mesentery. Small aggregates were also visible in the liver serosa, suggesting possible transportation to the liver. However, none of the tested nanoparticles affected the health of animals. This lack of toxic effect may suggest the potential applicability of nanoparticles as drug carriers for local therapies, ensuring accumulation and slow release of drugs into a targeted tissue without harmful systemic side effects.

## Introduction

Carbon is the most widespread element in all living organisms and thus carbon nanoparticles, due to their excellent biocompatibility, are an attractive alternative to traditionally used anticancer drugs and may be applicable in drug delivery systems.

We studied the biological properties of three different forms of carbon nanoparticles—diamond (DN), graphene oxide (GO) and graphite (GR). They differ in shape, proportion of chemical bonds, atomic hybridization (sp^2^ for GO and GR, and sp^3^ for DN) and functional groups; these factors affect their electrochemical and redox potential and influence their behavior in biological models [1–3]. DN, GO and GR have been reported to be non-toxic and biocompatible moieties [4–9], on the other hand showing antiproliferative and anticancer properties against tumor cells in *in vitro* models [10,11].

The excellent biocompatibility of DN was demonstrated has been *in vivo* studies on mice and rats, showing no toxic effects and no impact on the immune system [4,5]. DN has been also reported to be a good intracellular transporter of drugs like chemotherapeutics [12]. Moreover, DN have antiangiogenic properties, which lead to tumor growth inhibition [10], making DN a promising anticancer agent since angiogenesis is one of the major factors promoting tumor development. DN and GR, even in high doses, are non-toxic to developing chicken embryos, although the mRNA expression of basic fibroblast growth factor was downregulated and heart vascularization and vessel density were diminished [13]. Furthermore, in studies using a *Caenorhabditis elegans* (roundworm) model, GR similarly did not reveal any toxic effects [6]. Even exposure of rats to GR in inhaled air did not induce an inflammatory state in lungs [7]. GO is a relatively new nanomaterial, and has recently attracted interest in the biomedical field [2,14]. GO in a formulation with dextran was reported to be a stable and promising agent for MRI bioimaging [15]. GO is also considered as a new hope and future for regenerative medicine and tissue engineering since GO composites are ultra-strong, lightweight and can successfully reinforce biodegradable materials [16]. Different *in vitro* studies showed that graphene has anticancer and antiproliferative properties [17,18], and may have cytotoxic effect on normal, healthy cells [19], however, it depends on the form of used graphene, as well as on the production method. Similarly, antibacterial activity is also dependent on the form of used graphene [2]. Chowdhury et. al. [20] reported that GO functionalized with dextran had no adverse effect on hematological components *in vitro*, and did not induce endothelial dysfunction during *in vivo* studies with the hamster model. *In vivo* studies in mice, conducted by Yang et al. [8], demonstrated that GO has no toxic effects; however, they suggested that the administration route has an important impact on the final effect. The work of Ma-Hock et al. [7] contributed to this statement since, in their experiment, there was inflammation in the lungs of mice after GO exposure in inhaled air. Kanakia et al. [9] performed studies on rats, employing dextran-functionalized GO administrated intravenously in a wide range doses (1 to 500 mg/kg), demonstrating no effect on respiratory, cardiovascular or hematological indices at doses < 125 mg/kg. Minor changes in some organs (e.g. the liver) were reported, which were associated with GO particle accumulation. Longer exposure (30 days) showed only a negligible presence of GO in examined organs, suggesting removal and extraction through the feces.

Nevertheless, the majority of the *in vivo* results are based on short-term exposure to nanoparticles, by measuring acute responses, while the long-lasting effects have not been broadly investigated [5,21,22]. The excretion rate of DN, GO and GR is not certain since multiple reports have suggested potential accumulation of nanoparticles or their formulations in tissues [5,8,23–25]. Thus, it is essential to study the possible toxicity of prolonged carbon nanoparticle accumulation, which may remain in treated tissues for a long time after administration. Nanoparticle retention might be considered an advantage for creating drug delivery systems meant to stay within the target tissue, assuring the optimal drug concentration without harmful systemic reactions. The possible slow spread and circulating routes of nanoparticles should be also considered. The liver is the primary organ responsible for the accumulation of multiple substances, as it breaks down or modifies toxins, and is highly dependent on the oxidation capability of hepatocytes [26]. Therefore, the liver may be susceptible to damage caused by nanoparticles, which after accumulation, can be gradually passed to blood vessels and transported to the liver, triggering oxidative stress and inflammation [27].

We hypothesized that after multiple intraperitoneal injections, nanoparticles will be retained within the peritoneum, adhered to internal organs, and they will also penetrate into the liver since the majority of blood circulation within the peritoneal cavity, especially within mesentery, leads directly to the liver via veins connected to the hepatic portal vein. The hypothesis presumes long-lasting effects on animal health. The objective of this study was to evaluate systemic responses to the long-term exposure to bare nanoparticles. Consequently, we evaluated the growth and health status of rats after 4 weeks of injections followed up by a growth period of 8 weeks. The measurements included blood morphology and biochemical parameters, changes in oxidative status of the liver measured by total glutathione levels and lipid oxidation, as well as the inflammatory state assessed by C-reactive protein measurements and multiple cytokine levels.

## Material & Methods

### Ethics statement

The experimental procedures were performed in accordance with Polish legal regulations concerning experiments on animals (Dz. U. 05.33.289). The experimental protocols were accepted by the III Local Ethics Commission for Experimentation on Animals at Warsaw University of Life Sciences, Poland.

### Nanoparticles

ND and GR were obtained from Skyspring Nanomaterials Inc. (Houston, TX, USA). Both nanomaterials were produced by the explosive method and synthesized to 3–4 nm. According to the manufacturer, the purity was >95% for DN and > 93% for GR, and the specific surface area was 282 m^2^/g for DN and 540–650 m^2^/g for GR.

GO was obtained from the Institute of Electronic Materials Technology (Warsaw, Poland), produced by a modified Hummers method from graphite flakes (Asbury Carbons, Asbury, USA). The detailed data of the production method and a characteristic, including FTIR analysis, are presented in Kurantowicz et al. [2].

Pure powders of nanoparticles were suspended in sterile saline solution at a concentration of 500 mg/L and sonicated at 550 W/m^2^ for 1 h in an ultrasonic bath (Sonorex Super RK 514H, Bandelin Electronic, Germany) prior to injection.

#### Visualization of nanoparticles

**Fig 1:**
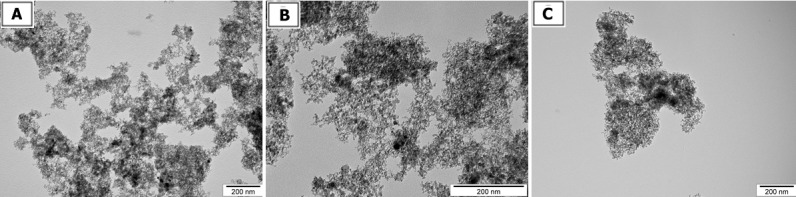
TEM pictures of diamond (A), graphene oxide (B) and graphite (C). Scale bar indicates 200 nm.

The size and shape of nanoparticles were inspected using transmission electron microscopy (TEM: JEM-2000EX; JEOL, Tokyo, Japan) at 80 keV. Images were captured with a Morada 11 megapixel camera (Olympus Soft Imaging Solutions GmbH, Münster, Germany). Droplets of nanoparticles solutions were placed onto formvar-coated copper grids (Agar Scientific, Stansted, UK) and immediately after air-drying the grids were inspected by TEM (Fig 1).

#### Zeta potential measurements

The Zeta potential of suspended nanoparticles were determined after 120 seconds of stabilization at 25°C by the Dynamic Laser Scattering electrophoretic method with Smoluchowski approximation by Zetasizer Nano-ZS90 (Malvern, Worcestershire, UK). Each measurement was repeated 3 times. The mean Zeta potentials of the DN, GO and GR solutions in 0.9% NaCl were -15.8 mV, – 8.80 mV and 12,5 mV, respectively (Table 1).

**Table 1:** An average size and Zeta potential of diamond (DN), graphene oxide (GO) and graphite (GR) nanoparticles. The size was estimated upon analysis by transmission electron microscopy. Zeta potential is presented as means ± standard deviation, n = 3.

	DN	GO	GR
Average size (nm)	3–4	8–25	3–4
Zeta potential (mV)	-15.8 ± 0.55	-8.8 ± 0.25	12.5 ± 0.43

### Animals

#### Animal maintenance

Forty female Wistar Crl:WI(Han) (strain ID number 2308816 in Rat Genome Database) outbred rats (6 weeks of age, average body weight [BW] 124 ± 19 g) were randomly divided into four equal groups (placebo, ND, GO, GR) and kept in separate cages for 4 to 12 weeks. The animals were housed in polycarbonate cages with steel wire tops. They were kept under standard conditions at a room temperature of 22 ± 2°C, 50–60% humidity and 12:12 light:dark cycle. The animals had free access to fresh water and dry pellet feed (Labofeed B standard, Morawski, Poland). Animal behavior, food and water intake and hair/skin condition were monitored.

#### Nanoparticle administration

Rats were administrated multiple intraperitoneal injections of 1 ml of physiological saline (0.9% NaCl) for the placebo group and 1 ml of the nanoparticle suspension in physiological saline for the remaining groups (ND group, GO group GR group). The injections were given for 4 weeks at three-day intervals (in total eight injections). The nanoparticle suspensions were given at a concentration of 500 mg/L, equivalent to 4 mg of nanoparticles/kg body weight. Some of the animals (seven from each group) were terminated 3 days after the last injection, while the rest (three from each group) were maintained for an additional eight weeks (12 weeks of the experiment in total) in order to evaluate the systemic response to long-term exposure to nanoparticles accumulated in the body.

#### Animal euthanasia

After 4 or 12 weeks, the animals were fasted for 12 hours then euthanized by the inhalation of isoflurane (Forane, USP, Baxter, Poland). Blood samples were collected from the heart by cardiac puncture (see below). Photographs of the general outlook of the rat interior were taken immediately after sacrifice. Livers were excised, washed with phosphate buffered saline, weighed and stored at -80°C for further analysis.

#### Blood morphology

One milliliter of blood was collected into tubes coated with K_3_-EDTA as an anticoagulant (Equimed, Krakow Poland). Complete blood was used for blood morphology examination, performed by automated hematologic analyzer (Abacus Junior Vet, Diatron Group, Budapest, Hungary). The following complete blood parameters were examined: number of white blood cells (WBC, 10^9^/L) and their subpopulation (all in 10^9^/L)—lymphocytes (LYM), granulocytes (GRA), monocytes (MID), number of red blood cells (RBC, 10^12^/L), plasma hemoglobin concentration (HGB, g/dL), hematocrit (HCT, %), number of platelets (PLT, 10^9^/L), relative distribution width of the red cell population (RDW, % of covariance), mean red blood cell corpuscular volume (MCV, fL), mean erythrocyte hemoglobin concentration (MCH, pg), mean cell hemoglobin concentration (MCHC, g/dL), mean platelet volume (MPV, fL), relative distribution width of the thrombocyte population (PDWc, in %) and plateletcrit (PCT, in %).

#### Blood serum biochemical indices

One milliliter of blood was collected into tubes with a clot activator (Equimed) for serum determinations. Serum was centrifuged at 3800 x *g* for 8 minutes at 4°C (Sorvall^®^ ST16, Thermo Fisher Scientific, Waltham, MA, USA). Biochemistry was performed at Clinical Chemistry Analyzer Miura One (I.S.E., Guidonia, Italy). All reagents essential for those analyses were purchased from Pointe Scientific Inc. (Canton, MI, USA). The following parameters were examined: aspartate aminotransferase (AST), alanine aminotransferase (ALT), alkaline phosphatase (ALP), glucose, creatinine, blood urea nitrogen (BUN), total protein (TP), albumins, lactate dehydrogenase (LDH), and triglycerides (TG).

### Liver evaluation

#### Liver tissue homogenate preparation

Tissue homogenates were prepared in the buffers provided by the manufacturer (Abcam^®^, Cambridge, UK), separately for each of the following analyses. The liver tissue was homogenized using TissueLyser LT (Qiagen, Germantown, MD, USA) with a pre-frosted adapter at 50 Hz for 10 minutes, followed by centrifugation at 13 000 x *g* for 10 minutes at 4°C (Sorvall^®^ ST16, Thermo Fisher Scientific). The supernatant (“homogenate”) was collected for analysis.

#### Total glutathione levels

Quantitative measurement of total glutathione levels was performed using a Glutathione Detection Assay Kit—Fluorimetric (Abcam^®^). The assay utilizes monochlorobimane (MCB), a dye that forms an adduct exclusively with glutathione. The dye fluoresces blue (Ex/Em = 380nm/461nm) when bound to glutathione of the reduced (GSH) or oxidized (GSST) form. The reaction is catalyzed by glutathione S-transferase. The GSH standard, which was provided in the kit, is used to prepare a standard curve to calculate the direct amount of total glutathione in samples.

The homogenate was prepared as described above (10 mg of liver tissue per 100 μl of Cell Lysis Buffer). Each sample was diluted 5 x prior to the measurement. Further steps were performed in accordance with the manufacturer’s protocol. Fluorescence measurements were performed employing an Infinite^®^ 200 PRO microplate reader with i-control™ Software (Tecan Group Ltd., Männedorf, Germany). Each sample was analyzed in triplicate.

#### Lipid peroxidation

Quantification of lipid peroxidation in liver tissue was performed using a Lipid Peroxidation (MDA) Assay Kit (Abcam^®^). Lipid peroxidation forms malondialdehyde (MDA) and 4-hydroxynonenal (4-HNE) as natural byproducts. The MDA in the sample reacts with thiobarbituric acid (TBA) to generate an adduct, which can be quantified colorimetrically (λ = 532 nm or fluorometrically (Ex/Em = 532/553 nm). The minimum detectable amount of MDA is 1 nmol/well and 0.1 nmol/well using the colorimetric and fluorimetric methods, respectively.

The homogenate was prepared as described above, in lysis buffer containing butylated hydroxytoluene (BHT) provided by the manufacturer (10 mg of liver tissue per 300 μl of MDA Lysis Buffer). Further steps were performed in accordance with the manufacturer’s protocol, including an additional step of filtering samples through a 0.22 μm syringe filter to eliminate turbidity. We performed both the colorimetric and fluorometric assays to choose the most accurate one. The measurements were performed employing an Infinite^®^ 200 PRO microplate reader with i-control™ Software (Tecan Group Ltd.).

#### C-reactive protein levels

Quantitative measurement of rat C-reactive protein (CRP) present in liver tissue was performed using an Enzyme-Linked Immunosorbent Assay kit (Abcam^®^). The provided CRP standard is used to prepare a standard curve to calculate the direct amount of the protein in samples. The minimum detectable amount of the protein for this assay is 0.7 ng/ml.

The homogenate was prepared as described above, in diluting buffer provided by the manufacturer (50 mg of liver tissue per 500 μl of buffer). The most suitable dilution of supernatant was determined empirically by performing a series of dilutions of pooled samples, in the range from 2 to 20 000. The 1:2000 dilution was chosen for this analysis since the detectable absorbance was within reader’s lower and upper limits. The assay was performed in accordance with the manufacturer’s protocol. Absorbance measurements were performed employing an Infinite^®^ 200 PRO microplate reader with i-control™ Software (Tecan Group Ltd, Männedorf, Switzerland). Each sample was analyzed in duplicate.

#### Analysis of multiple cytokines

For the evaluation of potential liver damage, we assessed the levels of multiple cytokines engaged in the inflammatory state, hypersensitivity, apoptosis and tissue regeneration. For this purpose, we used the rat Cytokine Antibody Array Membrane (Abcam^®^), prepared for the simultaneous detection of 34 cytokines. The following targets can be detected by this array: activin A, agrin, thymus chemokine-1, B7-2/CD86, CINC-1 (cytokine-induced neutrophil chemoattractant 1), CINC-2α (cytokine-induced neutrophil chemoattractant 2α), CINC-3 (cytokine-induced neutrophil chemoattractant 3), CNTF (ciliary neurotrophic factor), Fas ligand, fractalkine, GM-CSF (granulocyte macrophage colony-stimulating factor), ICAM-1 (intercellular adhesion molecule 1), IFN-γ (interferon γ), IL-1 R6 (interleukin 1 R6), IL-10 (interleukin 10), IL-13 (interleukin 13), IL-1α (interleukin 1α), IL-1β (interleukin 1β), IL-2 interleukin 2), IL-4 (interleukin 4), IL-6 (interleukin 6), leptin, LIX (LPS-induced CXC chemokine), L-selectin, MCP-1 (macrophages chemoattractive protein), MIP-3α (macrophage inflammatory protein 3α,), MMP-8 (matrix metalloprotease 8), β-NGF (β nerve growth factor), PDGF-AA (platelet-derived growth factor AA isoform), prolactin R, RAGE (receptor for advanced glycation end-products), TIMP-1 (metallopeptidase inhibitor), TNF-α tumor necrosis factor α), and VEGF (vascular endothelial growth factor). IgG is printed as a positive control.

The homogenate was prepared as described above, in diluting buffer provided by the manufacturer (50 mg of liver tissue per 500 μl of buffer). The total protein concentration (TPC) was determined using a Qubit^®^ 2.0 Fluorimeter with the Qubit Protein Assay Kit (Invitrogen, Paisley, UK). Afterward, three samples from each group (4 and 12 weeks of exposure time) were diluted and pooled to a final TPC concentration of 0.21 μg/μl. Then, membranes with printed antibodies were incubated overnight at 4°C with pooled samples, followed by a cocktail of biotin-conjugated antibodies (2 hours at RT), streptavidin labeled with HRP (overnight at 4°C), and finally detection buffer for chemiluminescence. All washes between incubation periods were carried out as indicated in the protocol. Incubation and wash steps were performed under gentle rotation at 150 rpm (Mini-Shaker PSU-2T, Biosan, Riga, Latvia). Chemiluminescence detection was performed using multiple exposure times (30 seconds to 5 minutes) with the ChemiDoc^®^ Imaging System with Quantity One Basic Software (Bio-Rad, Hercules, CA, USA).

### Statistical analysis

Data for glutathione, MDA and CRP levels were analyzed by two factorial analysis of variance (ANOVA) with the Bonferroni post-hoc test, where exposure time and nanoparticle treatment were considered as factors. For body weight gain and liver weight, blood morphology and serum biochemical indices, one-factorial ANOVA with Duncan post-hoc test was performed. All analyses were performed using Statgraphics^®^ Plus 4.1 software (StatPoint Technologies, Warrenton, VA, USA). Differences at *P* ≤ 0.05 were defined as statistically significant.

## Results

### General health status

We examined the systemic toxicity of diamond nanoparticles (DN), graphene oxide (GO) and graphite (GR) administrated intraperitoneally to rats for 4 weeks. The general health status of animals was evaluated after the last injection (4 weeks) and after an additional 4 weeks (totally 12 weeks). During the total period of the experiment, all rats behaved normally. No disturbing symptoms of toxicity were observed. Animals were eating and drinking normally and they were showed interest in an animal care person. All animals showed steady development and growth. None of the examined livers were abnormally developed. There were no significant differences in BW gain or liver weight between the groups (Table 2).

**Table 2:** Mean body weight gain (BW gain), shown as% of the initial body weight and mean liver weight (g/100g BW) in the groups treated with diamond (DN), graphene oxide (GO) and graphite (GR) nanoparticles.

		Placebo	DN	GO	GR	SE-pooled^1^	*P*-value
BW gain (%)	4 weeks	48.0	55.2	59.3	53.7	1.56	NS
	12 weeks	105.4	97.0	118.9	99.5	4.36	NS
Liver (g/100 g of BW)	4 weeks	4.22	4.26	4.34	4.58	0.271	NS
	12 weeks	3.28	3.12	3.20	3.48	0.308	NS

^1^Pooled standard error of means

### Animal dissection

Animal dissection did not reveal pathological features in the primary organs, and there were no signs of inflammation or tissue disruption in the macroscopic images. However, a substantial amount of nanoparticles was present in the body tissues of all treated groups. The nanoparticles formed spherical- or oblong-shaped aggregates, which were remarkable both in rats after 4 weeks of the experiment (data previously shown in Kurantowicz et al. [28]) and in rats after 12 weeks. No differences in the amount of aggregates were noted between treated groups.

**Fig 2:**
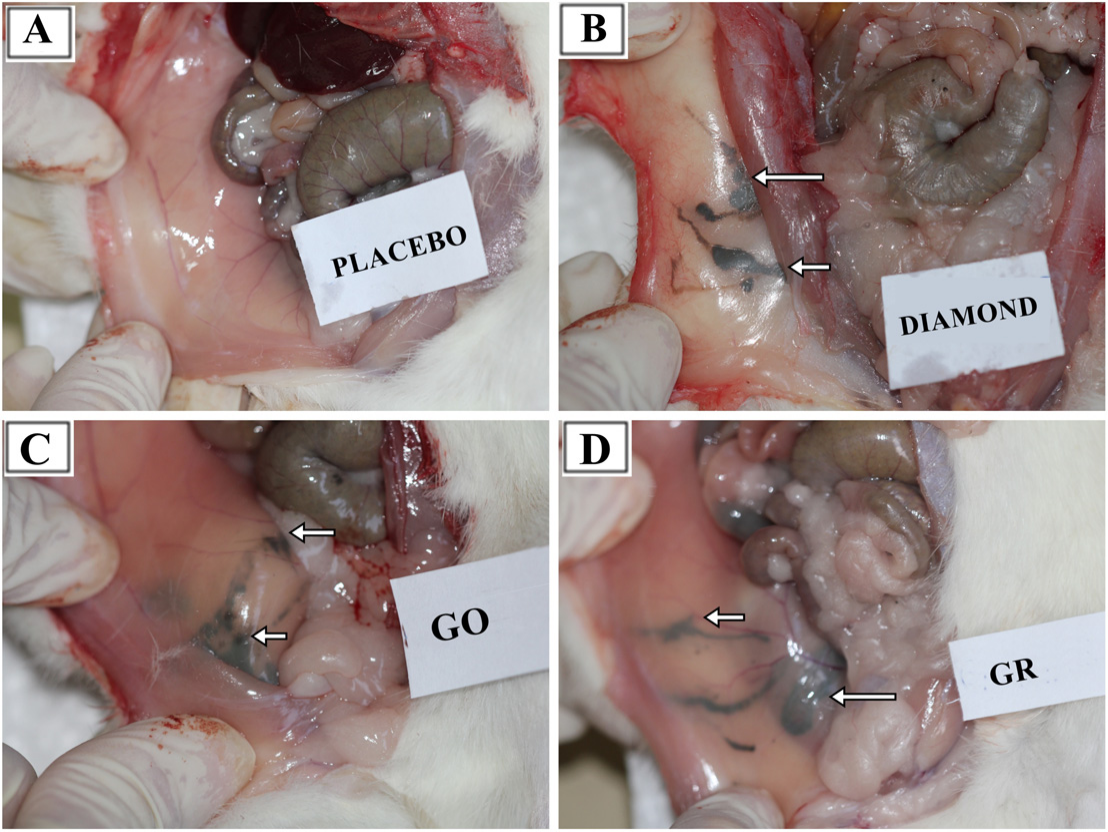
General view of dissected rat interior after 12 weeks exposure time. The right half of the rat abdomen is shown in the picture. **A**—Placebo, **B—**diamond, **C**—graphene oxide, **D**—graphite. Arrows indicate nanoparticle aggregates located between skin and abdominal muscles in the proximity of the injection.

**Fig 3:**
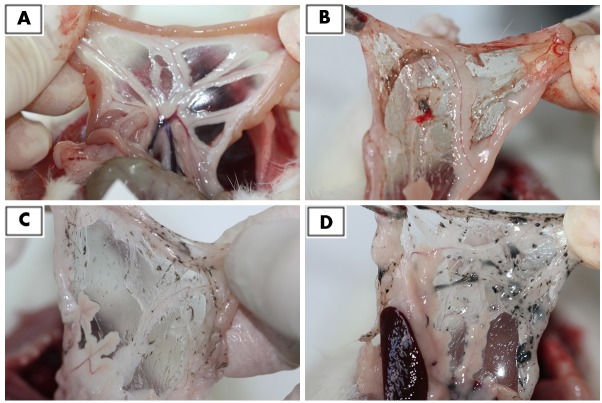
Mesenteries from dissected rats after 12 weeks exposure time, showing numerous small aggregates of nanoparticles. **A**—Placebo, **B—**diamond, **C**—graphene oxide, **D**—graphite. The most solid aggregates of nanoparticles are visible in the graphite group.

The most solid aggregates (up to 10 mm in diameter or length) were found in proximity to the injection site, between the connective tissues of the abdominal skin, muscles and peritoneum (Fig 2). Some of those aggregates spread in a radial manner. Numerous smaller, spherical aggregates were found among the mesentery (Fig 3), where the most solid aggregates were present in the GR group. Some of the smaller aggregates accumulated in abdominal lipid tissue (in the area of the injection). A few of the finest aggregates were present in the connective tissue surrounding liver.

### Blood morphology and serum biochemical indices

None of the examined parameters exceeded the reference values [29,30]. It should be noted that the range of reference intervals differs between authors and laboratories, and they are usually subjectively averaged from a variety of sources. All these parameters depend on the sex, age and strain of rat and laboratory methods, including sampling and testing techniques. Therefore, the range limits for the blood parameters of laboratory animals, which are presented in the literature, cannot be treated as strict values.

Mean red blood cell corpuscular volume (MCV) was the only blood parameter with statistically significant differences, with a higher MCV in DN group in comparison with placebo group (Table 3). However, mean values for both groups were within the reference interval.

**Table 3:** Results of blood morphology in rats after 12 weeks of exposure to diamond (DN), graphene oxide (GO) and graphite (GR) nanoparticles.

	Placebo	DN	GO	GR	SE—pooled^1^	*P*-value
**White blood cells (10** ^**9**^ **/L)**	2.28	1.66	2.23	1.94	1.306	NS
**Lymphocytes (10** ^**9**^ **/L)**	1.89	1.12	1.83	1.59	1.044	NS
**Monocytes (10** ^**9**^ **/L)**	0.06	0.09	0.04	0.06	0.068	NS
**Granulocytes (10** ^**9**^ **/L)**	0.33	0.44	0.36	0.30	0.302	NS
**Red blood cells (10** ^**12**^ **/L)**	7.33	7.14	7.47	7.31	0.796	NS
**Hemoglobin (g/dL)**	12.7	13.1	13.2	12.9	0.37	NS
**Hematocrit (%)**	37.2	39.2	39.5	37.8	3.81	NS
**Mean corpuscular volume(fL)**	50.7*	55.0*	53.3	52.0	1.56	0.0448
**Mean cell hemoglobin (pg)**	17.6	18.3	16.9	18.0	1.03	NS
**Mean cell hemoglobin concentration (g/dL)**	34.7	33.3	31.8	34.8	1.57	NS
**Red blood cells distribution width (%)**	17.4	17.5	17.5	16.9	0.72	NS
**Platelets (10** ^**9**^ **/L)**	509	422	309	540	175.7	NS
**Plateletcrit (%)**	0.33	0.28	0.22	0.39	0.127	NS
**Mean platelet volume (fL)**	6.50	6.37	7.10	7.00	0.647	NS
**Platelet distribution width (%)**	30.9	29.6	32.6	31.5	2.54	NS

^1^ Pooled standard error of means

*Value significantly different (*P*< 0,05) from Placebo group

As for the biochemical indices, there was a visible trend in the DN and GO groups for increased AST, ALT and LDH levels (Table 4), which are widely accepted as indicators of liver damage in different pathologic states. Nevertheless, the values remained within the reference ranges and were not significantly different.

**Table 4:** Serum biochemical indices in rats after 12 weeks of exposure to diamond (DN), graphene oxide (GO) and graphite (GR) nanoparticles.

	Placebo	DN	GO	GR	SE—pooled^1^	*P* -value
AST (IU/L)	249	347	292	112	185.7	NS
ALT (IU/L)	67.1	88.6	90.4	54.3	48.47	NS
ALP (IU/L)	132	89	120	160	36.2	NS
Glucose (mg/dL)	210	173	199	173	32.7	NS
Creatinine (mg/dL)	0.340	0.477	0.343	0.340	0.1491	NS
BUN (mg/dL)	36.9	48.4	41.5	35.7	7.64	NS
TP (g/L)	59.0	60.0	62.0	63.7	3.366	NS
Albumin (g/L)	51.7	51.0	50.7	55.3	3.24	NS
LDH (U/L)	865	1321	1384	845	494.7	NS
TG (mg/dL)	63.0	88.2	89.3	86.5	31.11	NS

AST—aspartate aminotransferase, ALT—alanine aminotransferase, ALP—alkaline phosphatase, BUN—blood urea nitrogen, TP—total protein, LDH—lactate dehydrogenase, TG—triglycerides

^1^ Pooled standard error of means

### Total glutathione

**Fig 4:**
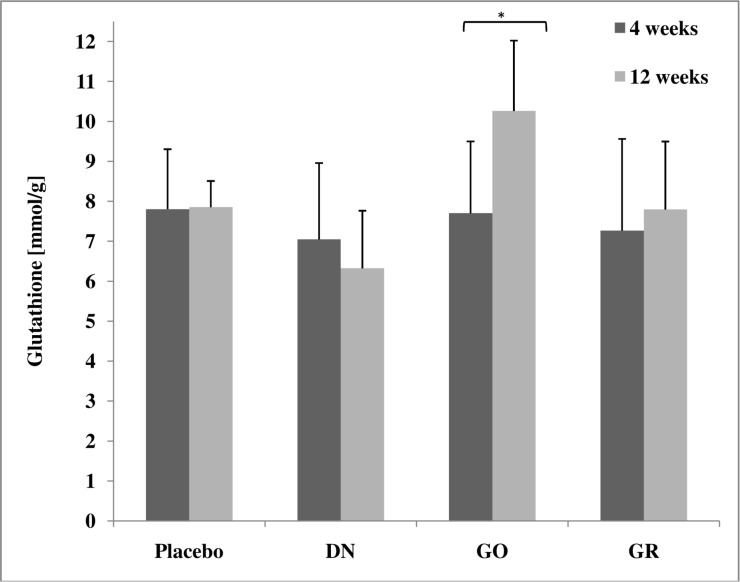
Concentration of total glutathione in rat liver (mmol/g of liver tissue). Results presented as mean **±** standard deviation (n = 3). * The value significantly (*P* < 0.05) differs from the placebo group.

There was significant interaction (*P*< 0.05) between exposure time (4 and 12 weeks) and treatment (placebo, DN, GO, GR). The total glutathione level in the GO group was significantly different (*P* < 0.05) than in the placebo group. The increase was especially visible in the liver tissue from animals kept for 12 weeks. A slight decrease in total glutathione was noticeable in the DN group; however, the difference was not significant (Fig 4).

### Lipid oxidation

**Fig 5:**
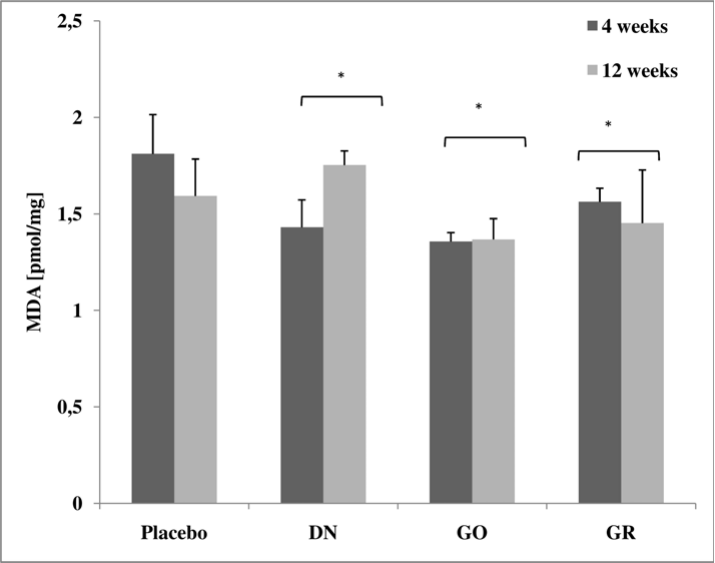
Concentration of malondialdehyde (MDA) in rat liver (pmol/mg of liver tissue). Results presented as mean ± standard deviation (n = 3). * The value significantly (*P* < 0.05) differs from the placebo group.

There was significant interaction (*P* < 0.05) between exposure time (4 and 12 weeks) and treatment (placebo, DN, GO, GR). The level of MDA in the DN, GO and GR groups was significantly lower (*P* < 0.05) than in the placebo group. In the DN group, the MDA level increased after 12 weeks of exposure in comparison to the level of MDA in animals sacrificed after 4 weeks (Fig 5).

### C-reactive protein

**Fig 6:**
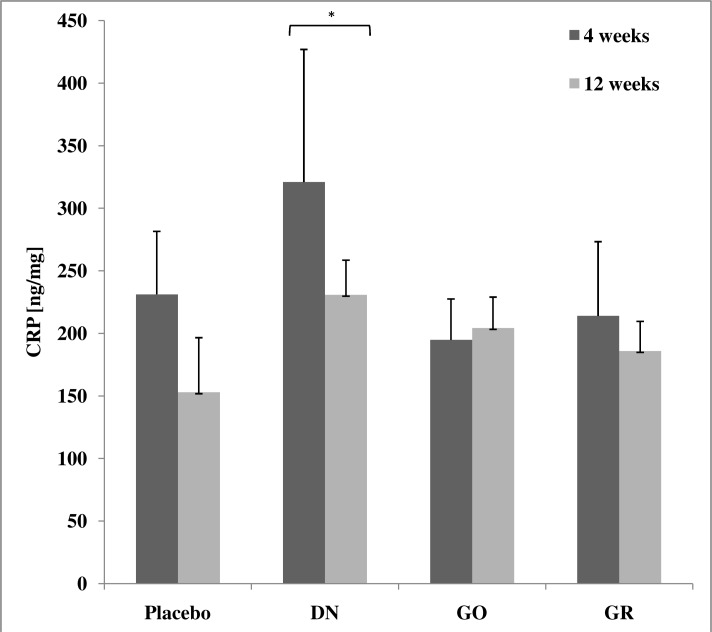
Concentration of C-reactive protein (CRP) in rat liver (ng/mg of liver tissue). Results presented as mean ± standard deviation (n = 3).* The value significantly (*P*< 0.05) differs from the placebo group.

There was no significant interaction between exposure time (4 or 12 weeks) and treatment (placebo, DN, GO, GR). The level of CRP in the DN group was significantly higher than in the placebo group (*P*< 0.05). In all groups, except GO, CRP decreased after 12 weeks of exposure in comparison to animals sacrificed after 4 weeks; however, these differences were not statistically significant (Fig 6).

### Cytokine levels

**Fig 7:**
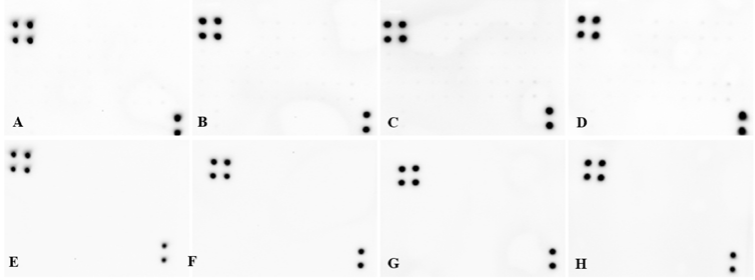
Rat cytokine membrane array used for cytokine detection in liver tissue. On each membrane, four spots in the upper left corner and two spots in the lower right corner represent IgG as a positive control. (**A, E**) Placebo; (**B, F**) diamond; (**C, G**) graphene oxide; (**D, H**) graphite. Upper row (**A, B, C, D**) - 4 weeks exposure, lower row (**E, F, G, H**) - 12 weeks exposure.

Analysis of liver tissue using the cytokine antibody membrane array did not reveal detectable expression levels of any of 34 cytokines connected with the inflammatory state, hypersensitivity, apoptosis or tissue regeneration. A strong signal from the IgG spots (four spots visible in the upper left corner and two in the lower right corner) served as a positive control of proper assay performance (Fig 7).

## Discussion

In the present experiment, the nanoparticles were injected intraperitoneally. The intraperitoneal route of administration is widely used for rapid testing of toxicity. This route of administration assures the fast absorption of a tested material, and the systemic availability is comparable to the intravenous route. The area of the peritoneal cavity is rich in lining and blood supply. Hence, the contact area assures a rapid absorption and potential fast toxic responses. Moreover, this route of administration is also useful for testing agents that are not soluble in water, like tested nanoparticles–they form a colloidal suspension, but not a water solution.

We assumed that nanoparticles injected intraperitoneally multiple times (at a dose of 4 mg/kg/BW per injection) would at least partially accumulate within the abdominal cavity and the tissues surrounding the site of injection and be transported into the blood circulatory system, and finally to different organs. According to data from the literature concerning the injection of functionalized GO, it is highly probable that most of the absorbed GO is accumulated in the liver [8,31]. Furthermore, the present results and our previous measurements (28) demonstrated an accumulation of nanoparticles in the liver serosa, which might imply that the particles were also retained in the liver. The liver is the main detoxifying organ, participating in storing, modifying and breaking down a broad range of substances. Many of these reactions depend on oxidation or reduction reactions. An imbalance in redox reactions leads to oxidative stress, which is the background for inflammatory and metabolic liver diseases [32]. Accumulation of nanoparticles may also lead to chronic hepatic fibrosis [22]. Thus, the liver is an organ that is highly exposed to damage, which could be caused by nanoparticles [33]. We decided to examine the oxidative state of the liver by evaluating biochemical serum indices, the level of total glutathione and lipid peroxidation, as well as possible inflammation by measuring C-reactive protein level and over-expression of 34 cytokines engaged in the inflammatory state, hypersensitivity and tissue regeneration.

The DN, GO and GR suspensions were given to animals in eight doses at three-day intervals. For four weeks of the nanoparticle administration, we constantly monitored the BW, behavior and possible signs of relapse, for example, extensive hair loss, poor water or feed intake, apathy or aggression. Animals grew and gained weight at a normal rate, comparable in all groups (Table 2), and their behavior remained friendly in terms of contact with a care person and no disturbing symptoms were noted. Three days after the last injection, we anesthetized some of animals and performed necropsy, which revealed substantial amounts of nanoparticles present in the abdominal wall and mesentery in the form of visible aggregates. Small aggregates were noticeable in fat tissue and connective tissue surrounding the peritoneal organs. This first insight into the animal body confirmed our previous presumption, that carbon nanoparticles may accumulate in an organism without immense toxic effects. We aimed to determine if DN, GO and GR remaining within body cavities for a longer period of time (an additional eight weeks after culling the first cohort) would induce toxicity, as well as if the aggregates would be still present in the abdominal wall or slowly excreted. So far, reports have mostly concentrated on the short-term effects of nanoparticles, in periods from a couple of hours, through days to 1 month [4,9,20,34]. There are only a few reports dealing with longer periods, from three [5,22] to five months [21]. Thus, the second cohort of animals was sacrificed after 12 weeks, what constitutes a significant part of a small rodent’s lifespan. Similar to 4 weeks of exposure [28], there was no mortality and no disturbing symptoms of relapse. Necropsy revealed that, after 12 weeks of the experiment, aggregates of DN, GO and GR were still present in large amounts within the abdominal wall (Fig 2) and mesentery (Fig 3). It should be noted that blood vessels in the mesentery of rats in DN group were not as prominent as in other groups. This might indicate another example of the influence of DN on blood vessels and angiogenesis, which has been demonstrated before [3,10]; however, the effect was not measurable. Altogether, the results suggest that local administration of selected carbon nanoparticles leads to significant accumulation within treated tissue, whereas other studies have shown that, for example, GO nanoparticles administrated intravenously are mostly excreted [9,20].

Blood analysis did not reveal obvious changes in the morphology parameters (Table 3) and all examined parameters were within range limits. Interestingly, increased levels of several enzymes in serum, usually connected with liver damage, were noticeable, although the differences were not significant. This trend was observed for aspartate transaminase (AST), alanine transaminase (ALT) and lactate dehydrogenase (LDH) in the DN and GR groups (Table 4). According to clinicians, the level should be magnified over five times to be considered as a marked increase [35]; therefore, the observed increase did not strictly confirm liver tissue damage.

Even though DN, GO and GR are all nanoparticles of carbon allotropes, they differ at the atomic level by the presence of various additional atoms (e.g. oxygen and hydrogen) and different hybridization of carbon atoms. In DN, there are mostly sp^3^ orbitals, assuring a strong, crystal form in the core, whereas in GO and GR there are mostly sp^2^ orbitals. The DN surface is rich in oxygen-containing groups (e.g. -COO and -OH), which makes DN suitable for introducing surface modifications, e.g. with bioactive molecules. Also, the large number of free electrons [36] makes DN a perfect donor of electrons, which may react with other radicals (e.g. reactive oxygen species, ROS), thereby acting as a radical scavenger. GO consist of a one-atom thick sheet, enriched with functional groups containing oxygen, like carboxylate (negatively charged), epoxide and hydroxyl groups (polar), which give GO hydrophilic features [37]. As well as, GO is rich in pi electrons and in sp^2^ orbitals, which are responsible for the hydrophobic character of these domains [37] and are characteristic for the graphene structure, although numerous of bonds are disrupted due to the introduction of large amounts of oxygen-containing groups [2]. We observed previously that this enrichment with oxygen significantly improves the water solubility and stability of the solution [2]. GR has almost exclusively sp^2^-bonded carbons and consists of multi-layered graphene sheets in the form of a crystal lattice, bound by weak van der Waals forces [38]. GR, in contrast to GO, is poorly covered with oxygen-containing groups and has extensively more pi electrons. All these differences cause differences in conductivity, redox potential and consequently reactivity with organic compounds in a living organism. A common feature of all carbon nanoparticles is the abundance of electrons, which may easily participate in redox reactions based on electron transfer.

Electrons in free radicals, bioactive substances and their metabolites (which have unpaired electrons) play an important role in physiological processes by the electron transfer phenomenon, which is generally a type of cell signaling [39]. Therefore, it should be expected that DN, GO and GR, which are rich either in unpaired electrons or in electron clouds of pi bonds, will have an effect on cellular physiology, provided that these nanoparticles have enough active surface. This condition is not exactly fulfilled when nanoparticles form large aggregates, because only the outer surface of the aggregate is in contact with the surrounding tissue, what could explain why we did not notice any signs of inflammation or tissue disruption across the abdominal wall, in spite of enormous amounts of nanoparticles, easily visible after 12 weeks.

Numerous small, spherical aggregates of nanoparticles were located within the mesentery, and a few of the finest aggregates were visible in the liver serosa. Mesentery blood vessels are connected to the superior mesenteric vein, which leads to the liver via the hepatic portal vein. Therefore, some portion of nanoparticles might be transported to the liver, which is prolific in electron transfer processes engaged in metabolism and detoxification pathways with redox state [40]. The presence of carbon nanoparticles in the liver after different administration routes has been reported before [8,33,41,42]. DN, GO and GR may then have an impact on liver function by interacting with redox reactions that maintaining the balance between production and scavenging of reactive oxygen species (ROS) [32]. The main compound of antioxidant defense against ROS and electrophiles is glutathione (GSH), which is responsible for a balanced oxidative state [43]. GSH contains active thiol (-SH), which is oxidized upon reaction with radicals and glutathione disulfide (GSSG) is created. GSSG can be reduced to GSH by the GSSG reductase, ensuring a constant balance between GSH/GSSG, where GSH should account for 98% of the total glutathione [43,44]. The liver plays a central role in GSH homeostasis [44]. We determined the total glutathione levels (GSH + GSSG) in rat liver after 4 and 12 weeks of the experiment, and the results showed a slight decrease in glutathione in the DN group (not statistically significant) and a significant increase in the GO group after 12 weeks (Fig 4). There was no effect in the case of GR. The results suggest the presence of GO nanoparticles in the liver tissue and accumulation at the molecular level in a time-dependent manner. The high concentrations of glutathione in the GO group may result from the characteristics of GO, structurally similar to quinones, which have carbon rings with pi electrons and functional groups rich in oxygen. GSH is the smallest antioxidant molecule in organisms and is able to not only react enzymatically but also to form non-enzymatic adducts with quinine-like particles, which are considered toxic to the organism [45]. Therefore, GSH might react similarly to GO particles. Furthermore, GO may behave like an oxidant and participate in electrophile generation; these are known to increase the level of GSH *via* several pathways, for example, the regulation of enzymes involved in GSH production [45]. GSH alterations are characteristic for pathological states. GSH depletion is associated with cellular death, cytotoxicity, an excess of radicals, oxidants and electrophiles, all of which demand *de novo* synthesis of GSH [46]. We hypothesize that increased levels of GSH may be a response to accumulating GO in the mentioned manner. However, most pathological states are associated with GSH deficiency, not excess [46].

Other evidence for the lack of oxidative stress in the liver was obtained with the evaluation of lipid peroxidation by measuring malondialdehyde (MDA) levels. MDA is one of the products of lipid oxidation and has been widely used as a marker for this process. Lipid peroxidation is caused by ROS and induces injury in cells, tissues and organs [47]. Interestingly, we observed that MDA levels were significantly lower in all the treated groups (DN, GO and GR), and was especially noticeable in the first cohort, culled after 4 weeks (Fig 5). This result may suggest that DN, GO and GR do not trigger oxidative stress and, more importantly, they may play a role in scavenging ROS, since the levels were lower than in the control (placebo) group. Exceptionally, in the DN group, there was a prominent increase in the MDA level after 12 weeks compared to the level after 4 weeks. This was likely due to DN accumulation; this large number of free electrons may behave in two ways, either by scavenging ROS or participating as ROS, depending on the concentration of DN in tissue.

Finally, we aimed to determine if DN, GO and GR triggered an inflammatory state in the liver. C-reactive protein (CRP) is a prototypical acute phase protein, which is produced by hepatocytes [48] in response to pro-inflammatory interleukins (IL). It is secreted into the circulating peripheral blood, and is a sensitive but non-specific marker of inflammation [49]. We measured the levels of CRP at the primary production site, showing a significant increase in the DN group after 4 weeks, but this was not different from the placebo group after 12 weeks (Fig 6). Since the increase was not time-dependent, it is unclear if CRP was increased because of DN accumulation or rather if it was due to administration itself since a similar situation was observed in the placebo group. The ultimate answer was obtained by the analysis of a number of cytokines using an antibody membrane array, which detects cytokines engaged in the inflammatory state, hypersensitivity, apoptosis and tissue regeneration (activin A, agrin, thymus chemokine-1, B7-2/CD86, CINC-1, CINC-2α, CINC-3, CNTF, Fas ligand, fractalkine, GM-CSF, ICAM-1, IFN-γ, IL-1 R6, IL-10, IL-13, IL-1α, IL-1β, IL-2, IL-4, IL-6, leptin, LIX, L-selectin, MCP-1, MIP-3α, MMP-8, β-NGF, PDGF-AA, prolactin R, RAGE, TIMP-1, TNF-α, and VEGF). The analysis ensures a robust and global insight into cytokine expression; moreover, the assay has excellent sensitivity. The signal detected from the positive control (IgG) confirmed proper assay performance, but we did not observe detectable expression of any of the studied cytokines. The membranes showed no differences between the DN, GO, GR and placebo groups (Fig 7). These results show that even when DN, GO and GR accumulated in the liver for a prolonged period of time, they did not trigger an inflammatory state.

These results clearly reveal that the tested carbon nanoparticles accumulate in significant amounts in proximity to the administration site for at least 12 weeks, and do not induce any noticeable toxicity in rats. The accumulation in the form of aggregates within peritoneum probably has prevented or reduced the systemic toxic effect. There was evidence that some of the nanoparticles were absorbed and transported to the liver, due to alterations in oxidative state parameters (GSH and MDA) and the CRP concentration; however, these changes were not detrimental. Our studies allow us to suggest that materials based on DN, GO and GR might be potential carriers in drug delivery systems, which are designed for local drug delivery to a targeted tissue without affecting the whole organism.

## Conclusions

In this study, we confirmed that DN, GO and GR are highly biocompatible and do not affect animal health after 12 weeks of exposure time. We showed that intraperitoneally injected nanoparticles formed aggregates in the vicinity of the injection site. The aggregates of nanoparticles injected during the first four weeks were present eight weeks later, but did not have any harmful effect on surrounding tissues. The nanoparticles could be transported to the liver, but they did not induce toxic effects. The results confirm the need for further studies on potential applicability of DN, GO and GR as drug carriers for local therapies, ensuring accumulation and slow release of drugs in a targeted tissue without harmful systemic side effects.
